# Body composition after allogeneic haematopoietic cell transplantation/total body irradiation in children and young people: a restricted systematic review

**DOI:** 10.1007/s11764-020-00871-1

**Published:** 2020-07-18

**Authors:** Ava Lorenc, Julian Hamilton-Shield, Rachel Perry, Michael Stevens, on behalf of the CTYA HSCT Adipose and Muscle Late Effects Working Group, Stephen Wootton, Martin Feelisch, Lars O. Dragsted, Marlou Dirks, Saeed Shoaie, Adil Mardinoglu, Helen Roche

**Affiliations:** NIHR Bristol Biomedical Research Centre (Nutrition Theme), Level 3, University Hospitals Bristol Education and Research Centre, Upper Maudlin Street, Bristol, BS2 8AE UK

**Keywords:** Stem cell transplantation, Leukaemia, Lipodystrophy, Sarcopenia, Adipose tissue

## Abstract

**Purpose:**

To collate evidence of changes in body composition following treatment of leukaemia in children, teenagers and young adults (CTYA, 0–24 years) with allogeneic haematopoietic stem cell transplant and total body irradiation (HSCT+TBI).

**Methods:**

Papers were identified by searching Medline and Google Scholar, reference lists/citations and contacting key authors, with no date or language restrictions. Inclusion criteria were as follows: leukaemia, HSCT+TBI, aged ≤ 24 years at HSCT and changes in body composition (total fat, central adiposity, adipose tissue function, muscle mass, muscle function). Quality was assessed using a brief Newcastle–Ottawa scale.

**Results:**

Of 900 papers, 20 were included: seven controlled, five uncontrolled studies and eight case reports. Study quality appeared good. There was little evidence of differences in total fat/weight for HSCT + TBI groups (compared to healthy controls/population norms/short stature controls). There was some evidence of significantly higher central adiposity and differences in adipose tissue function (compared to leukaemic/non-leukaemic controls). Muscle mass was significantly lower (compared to healthy/obese controls). Muscle function results were inconclusive but suggested impairment. Case reports confirmed a lipodystrophic phenotype.

**Conclusions:**

Early remodelling of adipose tissue and loss of skeletal muscle are evident following HSCT + TBI for CTYA leukaemia, with extreme phenotype of overt lipodystrophy. There is some evidence for reduced muscle effectiveness.

**Implications for Cancer Survivors:**

Body composition changes in patients after HSCT + TBI are apparent by early adult life and link with the risk of excess cardiometabolic morbidity seen in adult survivors. Interventions to improve muscle and/or adipose function, perhaps utilizing nutritional manipulation and/or targeted activity, should be investigated.

**Electronic supplementary material:**

The online version of this article (10.1007/s11764-020-00871-1) contains supplementary material, which is available to authorized users.

## Introduction

Leukaemia is the commonest type of cancer in children (0 to < 16 years) and one of the most common diagnoses affecting teenagers and young adults (16 to < 25 years). Patients who fail primary treatment, or those with very high risk factors at diagnosis, may be treated with allogeneic haematopoietic stem cell transplantation (HSCT) after conditioning with high dose chemotherapy and total body irradiation (TBI) [1]. Adult survivors of HSCT with TBI conditioning experience long-term morbidity, impaired quality of life and reduced life expectancy. Endocrine disorders including growth hormone deficiency, hypothyroidism and gonadal failure are well-described, but there is now good evidence of a phenotype emerging in early adult life that resembles accelerated ageing [2] with early post-transplant telomere shortening [3], long-term metabolic dysfunction [4], abnormal body composition [5], frailty [6] and fatigue [7]. Investigation has identified specific findings which incorporate features of the metabolic syndrome including hypertension, dyslipidaemia, insulin resistance, visceral adiposity and a pro-inflammatory state [8, 9].

Screening for adverse adiposity that increases cardiometabolic risk in the general population is relatively easy using standard measures of obesity (raised BMI and/or waist circumference) but is less straightforward in HSCT/TBI survivors who may not be overtly obese by these criteria. In contrast, the phenotype is characterized by the presence of increased visceral but reduced subcutaneous fat and reduced lean mass, i.e. they also demonstrate, at extremes, overt sarcopenic and lipodystrophic phenotypes [10]. These changes seem causally linked to the increased risk of metabolic syndrome in this patient population [1]. Metabolic syndrome has six components that relate to cardiovascular disease risk (based on the ATPIII definition): abdominal obesity, atherogenic dyslipidaemia, raised blood pressure, insulin resistance ± glucose intolerance, proinflammatory and prothrombotic states [11].

Survivors of all forms of cancer diagnosed as children or as teenagers and young adults (CTYA), including leukaemia treated without HSCT/TBI, may also face long-term morbidity in adult life depending on the nature of the treatment received; cardiovascular disease is the most common cause of early mortality in CTYA cancer survivors after the risk of death from second cancer [12]. Metabolic syndrome is also reported in other survivors of childhood cancer, but HSCT, TBI and cranial or abdominal irradiation all appear to incur greater risk [13]. Recent studies also confirm an increased risk of type 2 diabetes in adult survivors of childhood leukaemia [14].

The incidence, severity, progression and outcome of changes in body composition/BMI in survivors of HSCT/TBI undertaken in the CTYA age range are unclear. Nor is it known how their risk compares with survivors of CTYA leukaemia treated without HSCT/TBI or with individuals without a history of cancer treatment with or without evidence of obesity. Clarifying the phenotype of HSCT/TBI survivors may assist in developing future studies to investigate the critical pathophysiological changes that drive the associated cardiometabolic consequences likely to occur in adult life and in designing potential interventions.

## Aims

This restricted systematic review aimed to:

- collate evidence of changes in body composition/BMI in survivors of leukaemia treated in the CTYA age range (age 0–< 25 years) with HSCT with TBI
- identify evidence that body composition is associated with change in metabolic status in survivors
- describe dietary and exercise interventions used to ameliorate these changes in body composition
- compare findings, with studies of leukaemia survivors treated without HSCT with TBI and with the general population

## Methods

This review was registered on PROSPERO International prospective register of systematic reviews, reference number CRD42019138493. We followed Plüddemann’s framework for rapid reviews [15].

### Searches

Papers were identified by:

- Searching Medline via OVID using Medical Subject Headings (MeSH) and keyword terms (see Online Resource 1), with weekly email updates for papers published since the search. Medline was searched from its inception to the date of search (May 2019)
- Searching Google Scholar (first 20 pages of results) using search terms in Online Resource 1
- Contacting key authors (lead authors on included papers) to identify any work-in-progress or unpublished work
- Checking reference lists of and citations to key articles

### Study selection

Titles and abstracts were assessed for eligibility by AL, with RP independently assessing a random sample of 10% of records. Articles meeting inclusion criteria were retrieved in full and independently considered by two reviewers (MS, JHS). The reviewers resolved disagreements through discussion; reasons for excluding studies were recorded in a table.

The inclusion criteria were:

- Participants—we included studies of people:
  - Treated for all types of leukaemia with the addition of cases of non-Hodgkin’s lymphoma (NHL) and myelodysplastic syndrome (MDS) if included within a study of leukaemia patients
  - Treated with allogeneic HSCT and TBI (or both allogeneic and autologous if the allogeneic participants are analysed separately)
  - Aged up to and including 24 years (i.e. to 25th birthday) at HSCT
  - Any age at the time of evaluation
  - Studies including multiple conditions if leukaemia patients made up ≥ 90% of the sample or if results for leukaemia were analysed separately. Also, studies including patients treated with and without TBI if those with TBI made up ≥ 90% of the sample or results were analysed for TBI vs no TBI
- Comparators
  - Studies with or without a comparator
- Characteristics—studies which measured body composition changes, any of:
  - Sarcopenia (including impaired muscle strength)
  - Frailty (self-reported exhaustion, weakness (grip strength), slow walking speed, low physical activity and unintentional weight loss [16]
  - Lipodystrophy (abnormal fat distribution)
  - Changes in fat distribution, e.g. increased visceral/central fat
  - Changes in fat compartmentation/positioning
  - Body mass index (BMI)
- Intervention studies must use the intervention after the HSCT not before.
- Study design
  - Completed studies
  - With or without control groups
  - With or without interventions
  - Including case studies, feasibility studies, cohort studies
  - Literature reviews were only included in order to identify primary studies in their reference lists.

No date or language restrictions were applied.

Non-English papers were translated where possible.

### Data extraction

Full-text articles for inclusion were retrieved, and data extracted using a standardized data extraction template by AL, with RP independently extracting data from a random sample of 20% of articles and JHS and MS each independently extracting from a random sample of 10% of articles. Data extracted included the following: study methods (aim, setting, sample eligibility criteria, data collection methods and timing), participant flow (numbers eligible/recruited/followed up, reasons for non-participation), participant characteristics (diagnoses, treatment details, age at HSCT, age at follow up, sex, ethnicity) and outcome data (for each outcome, subgroup comparisons). The primary outcome data collected were:

- Total fat, e.g. BMI, whole body % fat
- Central adiposity, e.g. waist circumference, abdominal fat
- Adipose tissue function, e.g. adipokines, lipids
- Muscle mass, e.g. sarcopenia, frailty, lean body mass, fat-free mass
- Muscle function, e.g. muscle strength tests, frailty.

Secondary outcomes, only collected if body composition changes were also described:

- Measures of insulin resistance, glucose tolerance and metabolic syndrome

For studies which used interventions, we ensured adverse event data was extracted. The template was piloted before starting the review and modified as required to ensure consistency. Disagreements in opinion of data extracted were resolved through discussion.

### Quality assessment

To assess the quality of included studies, AL used a modified, brief Newcastle–Ottawa quality assessment scale [17]. Quality scores are reported in a table.

## Results

### Search results

**Fig. 1:**
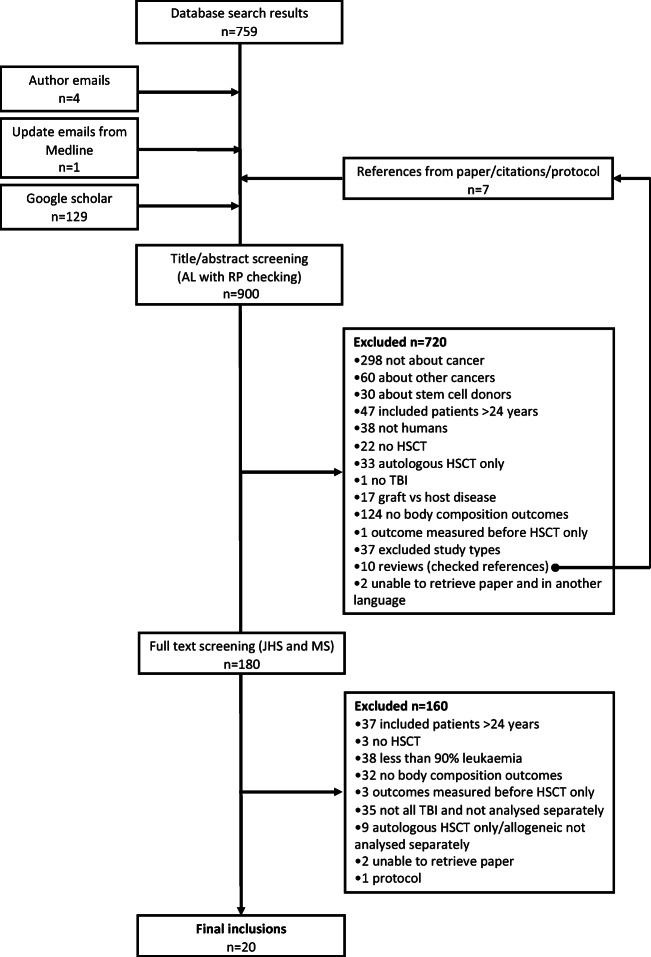
Flow chart to define literature search and study selection

Figure 1 details the search process. A total of 900 papers were identified, of which 880 were excluded. The most common reasons for exclusion were that studies were not about cancer or had no body composition outcomes (full reasons are given in Fig. 1). Of 24 emails to key authors, we received nine responses.

A final total of 20 papers were included—seven controlled studies [1, 10, 18–22], five uncontrolled studies [23–27] and eight case reports/series [28–35]. Only one study included an intervention [23].

Our exclusion criteria aimed to create a homogeneous set of papers relevant to a future study of interventions for body composition and frailty in childhood leukaemia HSCT with TBI. However, we are aware that some of the excluded papers may include relevant information so have provided these references in Online Resource 2.

### Study designs

Table 1 gives details of the twelve controlled and uncontrolled studies and outcome data are summarized in Table 2. ALL was the most common diagnostic group. Four studies included a range of diagnoses. Four of the seven controlled studies had two control groups and three studies only one. Controls included leukaemia patients without HSCT (5 studies), healthy people (3 studies) and other clinical groups (short stature or obese; 3 studies). Studies were conducted between 6 and 16.7 years after HSCT. Of our chosen body composition outcomes, eleven of the twelve studies measured total body fat, seven measured central adiposity and six measured adipose tissue function. Only four measured muscle mass and muscle function.

**Table 1:** Characteristics of studies (excluding case reports)

			HSCT patients					Control groups				Outcomes measured				
Author Year	Study design	Country	Diagnoses	*n*	Time since HSCT in years: median (range)	Age at HSCT in years: median (range)	Age at study in years: median (range)	Description	*n*	Matched to HSCT?	Same population as HSCT?	Total fat	Central adiposity	Muscle mass	Muscle function	Adipose function
Controlled studies																
Chow 2010 [18]	Cross sectional	USA	All ALL	26	6 (1–13)	NR	15 (8–21)	Leukaemia; chemotherapy only	55	No	Yes	Y	Y	N	Y	Y
Davis 2015 [19]		UK	16 ALL, 4 AML, 2 CML	22	8.8(1.4–19.2)	NR	6–24.5	Short stature	19	No	Same hospital	Y	Y	Y	N	N
Mostoufi-Moab 2015 [1]		USA	13 ALL, 7 AML, 2 JML, 2 aplastic anaemia, 1 CML	25	9.7 (4.3 to 19.3)	8.5 (0.4 to 18.3)	17.3 (12.2 to 25.1)	Healthy controls	25	Yes	No	Y	Y	Y	Y	Y
Nysom 2001 [20]	Retrospective cohort	Denmark	21 ALL, 2 AML, 1 CML, 1 NHL	25	8 (4–13)	NR	NR	Leukaemia; chemotherapy only	95	No	Yes	Y	N	N	N	N
								Healthy controls	463	No	No					
Taskinen 2013 [21]		Finland	All ALL	34	NR	NR	12.0 (9.0–30.0)	Leukaemia; chemotherapy only	45	No	Yes	N	N	N	Y	N
								Healthy controls	522	No	No					
Wei 2017 [22]		UK	All ALL	21	NR	9.5 (3.0–17)	21.4 (16.1–26.2)	Leukaemia; chemotherapy only	31	No	Yes	Y	Y	N	N	Y
								Obese young adults	30	No	Same hospital					
Wei 2015 [10]		UK	All ALL	21	NR	9.3 (2.6–16.7)	21.0 (16.1–26.1)	Leukaemia; chemotherapy only	31	No	Yes	Y	Y	Y	N	Y
								Obese young adults	30	No	Same hospital					
Uncontrolled studies																
Adachi 2017 [26]	Cross sectional	Japan	18 ALL, 10 AML, 1 ML (we analysed only leukaemia)	29 (leuk only)	NR	5.9 (1.0–14.2)	15.6 (7.0–27.5)	From additional data, we identified a potential control group of *n* = 3 patients treated without TBI but this was not a controlled study.				Y	Y	N	N	N
Inaba 2012 [27]	Retrospective cohort	USA	68 AML, 61 Lymphoid, 33 CML, 17 MDS	179	6.6 (1.0 to 17.7)	11.3 (2.1 to 21.3)	NR					Y	N	Y	N	N
Davis ND [23]	Before and after	UK	NR	21	16.7 (10.9–24.5)	NR	16.7 (10.9–24.5)					Y	N	N	Y	N
Freycon 2012 [24]		France	39 ALL, 4 AML, 4 CML 2 MDS	49	14.4 (4.5–21.9)	10.5 (2.3–17.4).	24.3 (18.9–35.8)					Y	N	N	N	Y
Chemaitilly 2009 [25]	Non comparative	USA	7 ALL, 3 AML	10	NR	13.0 (8.6–19.6)	24.0 (18.0–30.3)					Y	Y	N	N	N

*NR* not reported

**Table 2:** Outcome data

Outcome	Study groups							*P* value (significant values in italics)	Association with metabolic syndrome and/or gender	Ref
	1. Leukaemia + HSCT + TBI		2. Leukaemia no HSCT		3. Non-leukaemic controls					
	*n*	results	*n*	Results	Description	*n*	Results			
Total fat										
BMI (mean ± SD or median (range))	25	19.8 (15.3 to 34.4)			Matched healthy controls	25	22.4 (18.0 to 28.0)	NS		[1]
	25	19.7	95	NR	National reference values		19.8	*1 v 2 0.007* *1 v 3 CI: − 0.69 to 0.33*		[20] [20]
					Healthy controls	463	NR	*0.04*		
	47	[single group] At diagnosis 16.7 ± 2.7; at TBI 17.6 ± 2.8; at follow-up 20.5 ± 4.1						TBI v follow-up: *0.003* for boys NS for girls	Weight loss greater for males	[24]
	29	16.81 ± 3.10	Leukaemia no TBI					Control group too small to calculate significance		[26]*
			3	17.95 ± 1.97						
BMI *z* scores/SDS	26	0.80 ± 0.92	48	0.54 ± 1.29				0.31		[18]
	25	− 0.28 (− 2.94 to 2.22)			Matched healthy controls	25	0.44 (− 1.42 to 1.72)	NS	No sex difference	[1]
	18 (all had GHD)	− 0.07 (1.56)			Short stature normal GH	12	− 0.42 (1.81)	NS		[19]
	21	− 0.4 (2.0)	31	1.0 (1.3)	Obese young adults	30	3.2 (0.6)	*Overall group diff < 0.001* *1 v 2 0.001* *1 v 3 < 0.001*		[10]
	16	[Single group] Timepoint 1: − 0.07 (1.63), Timepoint 2: − 0.08 (1.53), Timepoint 3: − 0.28 (1.68), Timepoint 4: − 0.40 (1.79)						*ANOVA: 0.07* *Time 2 vs 3 0.025*		[23]
Obese BMI > 30 (*n*)	21	2 (10%)	31	6 (19%)	Obese young adults	30	30 (100%)	1 v 2 0.45 1 v 3 *< 0.001*		[22]
	10	3							2/3 were female	[25]
Overweight BMI 25< >29.9	10	2							2/2 were male	[25]
Overweight or obese	179	[Single group] Before HSCT 30.1%, 10 years post-HSCT 23.0%						*< 0.001*		[27]
Underweight	179	[Single group] Before HSCT 4.5%, 10 years post-HSCT 11.5%						*< 0.001*		
Whole body fat mass (kg)	25	14.8 ± 8.6			Matched healthy controls	25	12.7 ± 3.6	0.26	Associated with insulin resistance in HSCT participants	[1]
Whole body fat mass *z* score	25	0.72 ± 1.06			Matched healthy controls	25	0.37 ± 0.75	0.19		
	25	0.72 ± 1.06			Reference controls	1001	NR	*0.001*		
	179	[Single group] Before HSCT NR but close to population mean of 0.31, 10 years post-HSCT − 0.27						0.083	Higher in females (*p* = 0.002)	[27]
Body fat %	18 (all had GHD)	32.9 (11.1)			Short stature normal GH	12	22.4 (10.3)	*0.039*		[19]
	16	[Single group] Timepoint 1: 31.1 (15.0), Timepoint 2: 29.5 (13.9), Timepoint 3: 28.3 (14.0), Timepoint 4: 26.6 (13.2)						ANOVA: 0.27 Pairwise NS	Higher in females	[23]
	25	24.8%	95	?	Healthy controls	463	19.1%	1 v 2 estimated difference 0.11 *z* score, CI 20.46 to 0.68, *P =* 0.70 1 v 3 *z* = 1.05, CI 0.56 to 1.54, *P = 0.0002*	Not significantly related to sex	[20]
Fat mass index (FMI)	18 (all had GHD)	5.72 (1.77–14.05)			Short stature normal GH	12	3.41 (1.33–11.01)	NS	Associated with gender (across all groups) but not pubertal status	[19]
High fat mass index on DEXA (> 95th centile) (normal population references = 5%)	21	9 (43%)	30	17 (57%)	Obese young adults	21	21 (100%)	1 v 2 0.57 (0.17–1.76) *P* = 0.33 *1 v 3 0.018 (< 0.01–0.33) P = 0.007*		[10]
Central adiposity										
Waist-to-hip ratio	26	0.83 ± 0.07	48	0.90 ± 0.068				*< 0.01* (including after adjustment for age and sex)		[18]
	18 (all had GHD)	0.94 (0.06)			Short stature normal GH	12	0.96 (0.05)	NS		[19]
	21	0.9 (0.09)	31	0.84 (0.08)	Obese young adults	30	0.93 (0.08)	*Overall group diff < 0.001* *1 v 2 0.003* 1 v 3 0.76 *2 v 3 < 0.001*		[10]
High waist-to-hip ratio (M > 0.9, F > 0.85) (*n*)	21	13 (62%)	31	12 (39%)	Obese young adults	30	25 (83%)	1 vs 2 *0.007* 1 v 3 1.0	Associated with metabolic syndrome	[22]
	10	6								[25]
Waist circumference	29	64.1 ± 9.80	Leukaemia no TBI					Control group too small to calculate significance		[26]*
			3	68.9 ± 4.7						
Waist circumference SDS	18 (all had GHD)	1.00 (1.62)			Short stature normal GH	12	− 0.26 (1.58)	*0.043*		[19]
Waist–height ratio	18 (all had GHD)	0.49 (0.07)			Short stature normal GH	12	0.45 (0.06)	NS		[19]
	21	0.5 (0.07)	31	0.5 (0.08)	Obese young adults	30	0.7 (0.07)	*Overall group diff < 0.001* 1 v 2 0.87 *1 v 3 < 0.001*		[10]
High waist height ratio (> 0.5) (*n*)	21	10 (48%)	31	17 (55%)	Obese young adults	30	30 (100%)	1 v 2 0.61 *1 v 3 < 0.001*		[22]
	10	6								[25]
High waist circumference (≥ 90th percentile for age and sex, ≥ 80 cm in females and ≥ 94 cm in males) (*n*)	26	13 (27.1%)	48	7 (26.9%)				1.00		[18]
	21	6 (29%)	31	16 (52%)	Obese young adults	30	30 (100%)	1 v 2 0.10 *1 v 3 < 0.001*		[22]
Trunk fat %	18 (all had GHD)	33.4 (13.4)			Short stature normal GH	12	22.2 (10.3)	*0.041*		[19]
Trunk fat mass index (kg/m^2^)	21	4.2 (2.3)	30	8.9 (4.3)	Obese young adults	21	16.4 (3.3)	*Overall group diff < 0.001* 1 v 2 0.31 *1 v 3 < 0.001*		[10]
Visceral fat %	25	55.6 (4.6 to 166.7)			Matched healthy controls	25	43.8 (15.9 to 75.1)	*< 0.01 (adjusted for sex)*	Associated with insulin resistance in HSCT participants	[1]
	20	15.5 (6.2)	30	12.1 (4.9)	Obese young adults	21	11.7 (2.6)	*Overall group diff 0.023* *1 v 2 0.047* *1 v 3 0.043*		[10]
Visceral fat to total fat (%)	20	26.6 (8.3)	30	20.6 (7.9)		30	15.8 (3.7)	*Overall group diff < 0.001* *1 v 2 0.012* *1 v 3 < 0.001*		
Visceral-to-subcutaneous fat ratio > 0.4	20	12 (60%)	30	6 (24%)		21	0 (0%)	*1 v 2 0.002* *1 v 3 0.003*		
Android fat (%)	21	41.0 (14.0)	30	40.1 (12.7)		21	55.6 (5.6)	*Overall group diff < 0.001* 1 v 2 0.98 *1 v 3 = 0.001*		
Gynoid fat (%)	21	38.4 (10.6)	30	42.6 (11.4)		21	51.4 (6.4)	*Overall group diff < 0.001* 1 v 2 0.20 *1 v 3 < 0.001*		
Android-to-gynoid fat ratio	21	1.1 (0.2)	30	0.9 (0.2)	Obese young adults	21	1.1 (0.1)	*Overall group diff = 0.001* *1 v 2 0.015* 1 v 3 0.78		
Adipose tissue function										
Adiponectin	25	8407 (2091 to 17,056) (ng/mL)			NR				Associated with insulin resistance in HSCT participants	[1]
	20	3.1 (1.3–10.3) (mcg/mL)	29	5.8 (2.9–20.2)	Obese young adults	21	4.2 (1.7–7.8)	*Overall group diff < 0.001* *1 v 2 < 0.001* 1 v 3 0.055		[10]
	26	− 0.32 (− 0.52 to 0.13) (multivariate regression estimate)	48	Reference group in regression (Adjusted for sex, current age, race/ ethnicity, and institution)				*0.01*	Decreased adiponectin only seen in those with insulin resistance.	[18]
Leptin	26	1.01 (0.55 to 1.46) (multivariate regression estimate)	48					*< 0.01*		
Triglycerides median (range), mg/dL	26	127 (63–327)	48	63 (16–177)				*< 0.01*		[18]
Raised triglycerides (> 1.7 mmol/L)	21	10 (48%)	31	3 (10%)	Obese young adults	30	4 (13%)	*1 v 2 0.004* *1 v 3 0.011*		[22]
HDL, median (range), mg/dL	26	45 (32–63)	48	54 (33–108)				*< 0.01*	Decreased HDL only seen in those with insulin resistance.	[18]
Low HDL (M < 1.03 mmol/L, F < 1.29 mmol/L)	21	12 (57%)	31	8 (27%)	Obese young adults	30	16 (53%)	*1 v 2 0.028* 1 v 3 0.79	Remained significant after correction for gender (CI − 0.16 to − 0.01, *p* = 0.023).	[22]
Muscle mass										
Fat-free mass index (kg/m^2^)	21	13.9 (2.4)	30	15.4 (2.3)	Obese young adults	21	17.5 (2.0)	*Overall group diff < 0.001* 1 v 2 0.066 *1 v 3 < 0.001*		[10]
	18	12.67 (1.69)			Short stature normal GH	12	15.43 (3.50)	*0.008*		[19]
Low fat-free mass index^1^ (< 5th centile) (normal population references = 5%)	21	15 (71%)	30	12 (40%)	Obese young adults	21	1 (5%)	1 v 2 *0.003* 1 v 3 < *0.001*		[10]
Muscle density (g/cm^3^)	25	75.8 (72.2 to 77.9)			Matched healthy controls	25	76.4 (75.0 to 77.6)	*0.04*		[1]
Whole body lean mass (kg)	25	35.6 ± 11.3			Matched healthy controls	25	46.0 ± 10.8	*< 0.001*		
Whole body lean mass *z* score	25	− 0.88 ± 1.28			Matched healthy controls	25	− 0.18 ± 0.75	*0.04*		
					Reference controls	1001	NR	*< 0.001*		
Leg lean mass (kg)	25	12.4 ± 4.1			Matched healthy controls	25	16.7 ± 4.1	*< 0.001*		
Leg lean mass *z* score	25	− 1.44 ± 1.49			Matched healthy controls	25	0.00 ± 0.85	*< 0.001*		
Lean mass/height^2^ mean *z* score	134	[Single group] Before HSCT − 0.30, 10 years post-HSCT − 1.26						*0.018*	Lower in females (*p* = 0.013)	[27]
Muscle function										
Physical activity	26	− 1.62 (− 2.90 to − 0.34)	48	Reference group in regression (Adjusted for sex, current age, race/ ethnicity, and institution)				*0.01*		[18]
	25	2.2 ± 0.8			Matched healthy controls	25	2.4 ± 0.5	0.48		[1]
Leg-lift	34	− 0.3 (1.0)	45	− 0.3 (1.5)				NS		[21]
Repeated squatting		− 0.3 (1.2)		− 0.6 (1.3)				NS		
Sit-up		− 0.2 (1.3)		− 1.8 (1.6)				*< 0.001*		
Sit and reach		0.3 (0.9)		− 1.0 (1.5)				*< 0.001*		
Back extension		− 0.5 (1.0)		− 1.1 (1.1)				*0.008*		
Shuttle run		− 0.5 (1.9)		− 1.3 (1.8)				*< 0.001*		
Muscle sum score		− 0.3 (1.1)		− 1.0 (1.2)				*< 0.001*		
Strength	16							Significant gains in strength were identified from 1RM data as follows: 81.5 (40.4)% increase in leg strength *(P < 0.001)* and 90.4 (78.9)% increase in chest strength *(P < 0.001)*.		[23]

*NS* not significant, *NR* not reported

*Values for Adachi et al. [26] were calculated for this review from datasets sent by the author

The eight case reports/series are presented in Table 3, representing a total of eleven cases.

**Table 3:** Case reports/series

Author Year	Details of case								Body composition results*				
	Diagnosis	Demographics	Time since HSCT (years)	Age at HSCT (years)	Age at study (years)	Treatment for leukaemia	Other diagnoses	Other treatments	Total fat	Central adiposity	Muscle function	Adipose tissue function	Association of metabolic syndrome and body composition
Amin 2001 [35]	ALL	16 years old female, Caucasian	10	6	16	Chemotherapy, craniospinal radiation, HSCT	Growth hormone deficiency at 9.5 years. Ovarian cyst at 14 years. Focal nodular hyperplasia. Bilateral cataracts.		BMI 18.8 kg/m^2^			Dyslipidaemia deteriorated progressively over 2 years.	
Ceccarini 2017 [28]	AML	20 years old female	11	9	20	Polychemotherapy, body irradiation, autologous HSCT.	Chronic GVHD. Diabetes type 2. Fatty liver disease. GH deficient. Hypothyroid. Hypogonadism.	Immunosuppressive treatment and photopheresis for GVHD.	BMI 14 kg/m^2^	Reduced subcutaneous fat at the limbs and gluteal region whilst she had preserved fat in the cheeks with a puffy appearance. Reduced amounts of fat in the legs (16%), increased % fat trunk/% fat legs (1.67) and trunk/limb fat mass (1.43)		Total cholesterol 277 mg/dL. Triglycerides 654 mg/dL. Serum leptin 7.4 ng/mL	
Kimura 2017 [29]	ALL	10 years old, female	6	4	10	HSCT	GVHD, hyperglycaemia and elevated haemoglobin A1c.	Treatment for GVHD. See Table 1 in the paper for full medication list.	Weight 19.5 kg (< 3rd percentile, − 3.65 SDS; height not measured due to contractures)	Full cheeks, distended abdomen, thin extremities without subcutaneous fat. Clinical diagnosis of acquired partial lipodystrophy (based on physical and computed tomography imaging findings).	Limited range of motion, poor muscle tone. Contractures bilaterally	Hepatic fatty changes detected on imaging. Hypertriglyceridemia	When hypertriglyceridemia improved, total daily insulin requirement decreased by approximately 25%. With continued improvement, insulin discontinued and resumed when triglyceride level increased.
Rajendran 2013 [30]	ALL	20 years old, female, Caucasian	14	6	20	UKALL-XI [5] protocol HSCT	Ovarian failure, mild bilateral cataracts, osteopaenia, (12 years old). Type 2 diabetes, hypertension and dyslipidaemia (since 15 years old). Endometrial atrophy, cervical fibroids, total abdominal hysterectomy, bilateral salpingo-oophorectomy (19 years old). Severe hypertriglyceridaemia, eruptive xanthoma and acute pancreatitis (on presentation).	Plasmapheresis and intravenous insulin (for pancreatitis). Various medications (see page 240). Intravenous insulin and subcutaneous heparin therapy. Dietary advice.	BMI 23.14	Adipose deposition more pronounced centripetally.		On presentation: Total cholesterol 29 mmol/L, Serum triglycerides 300.9 mmol/L. 2 months later: total cholesterol 6.7 mmol/L, triglycerides 3.5 mmol/L.	
Rooney and Ryan 2006 [31]	ALL	14 years old, female		14	NR	High-dose cyclophosphamide (60 mg/kg) and TBI. HSCT.	Sclerodermatous chronic GVHD. Diabetes.	Treatment for GVHD. Hormone replacement therapy. Dietary advice. Gliclazide therapy and fenofibrate. Insulin.	BMI 21.1	Lipodystrophy affecting mainly legs, thighs, buttocks and forearms. Waist circumference 72 cm, hip 70 cm, ratio 1.0. After 24 weeks of combination treatment, body weight had increased slightly (basal 54.2 kg, 19 weeks 55.4, 24 weeks 54.6 kg) but no significant change in waist–hip ratio (basal 1.0, 19 weeks 1.0, 24 weeks 1.0).		Fasting triglycerides 14.7 mmol/L. Cholesterol 5.9 mmol/L. HDL cholesterol 1.0 mmol/L. Liver function tests were normal. After 24 weeks of combination treatment no significant increase in serum adiponectin (basal 0.90 μg/mL, 19 weeks 0.41 μg/mL, 24 weeks 0.71 μg/mL) and no improvement in glycaemic control (basal HbA1c 8.7%, 19 weeks 9.3%, 24 weeks 9.3%).	Lipodystrophy was associated with hypertriglyceridaemia and insulin-resistant diabetes.
Adachi 2013 [32]	AML	Female	NR	NR	18	3 HSCTs.	GVHD, chemotherapy-related leukoencephalopathy, intractable epilepsy	Steroid therapy	BMI 17.7	Lipodystrophy (estimated onset aged 11 years). Remarkable abdominal distension- abdominal circumference 69 cm (navel level). Both extremities and buttocks showed marked reductions in subcutaneous fat.		Dyslipidaemia evident. Fasting triglyceride levels of 675 mg/dL, high-density lipoprotein cholesterol of 39 mg/dL and low-density lipoprotein cholesterol of 168 mg/dL	
	AML	Female	NR	NR	21	HSCT	GVHD, neck necrosis, aplastic anaemia following parvovirus infection, multiple hepatic angiomas.	Immunosuppressants	BMI 12.2	Lipodystrophy (estimated onset 13 years). Abnormal fat distribution (age 15)		Dyslipidaemia and fatty changes in the liver.	
	ALL	Male	NR	NR	23	2 HSCTs	GVHD	Immunosuppressants. Growth hormone.	BMI 16.5.	Lipodystrophy (estimated onset 12 years). Abnormal pattern of subcutaneous fat distribution (age 19).		Dyslipidaemia and hyperinsulinism. Fatty changes in the liver.	
Hosokawa 2019 [33]	AML	12 years old, female	10	21 months	12	HSCT	GVHD (acute then chronic). APL with metabolic disease after HSCT	Chemotherapy at 7 months. 3 years prednisolone for GVHD	BMI 13.2 (SD: − 4.1) kg/m2	Waist circumference 55 (SD: − 1.4) cm Waist circumference ratio 0.40 (SD: +0.45)		Fasting triglyceride levels 332 mg/dL. HDL-C 33 mg/dL Adiponectin 1.6 μg/mL Leptin 5.6 ng/mL	
	ALL	17 years, female	9	8	17	HSCT	Acute GVHD	3 years prednisolone for GVHD	BMI 17.0 (SD: − 2.0) kg/m2	Acquired partial lipodystrophy. Rather abundant subcutaneous fat in her cheeks and neck but lacked fat tissue in the upper and lower extremities and the gluteal region.		Fasting triglyceride levels 927. HDL-C 34. Adiponectin < 1.9 μg/mL. Leptin 3.5 ng/mL.	
Mayson 2013 [34]	ALL	22 years old, female	15	7	22	Chemotherapy for ALL at 3 to 6 years. HSCT for central nervous system relapse aged 7.	Uncontrolled insulin resistant type 2 diabetes (diagnosed aged 16) and severe hypertriglyceridemia. GVHD (resolved aged 11). Bilateral cataracts, short stature, and secondary oligomenorrhea.		BMI 22.4	Central adiposity, no frank lipoatrophy		Free fatty acids elevated despite hyperinsulinemia. Leptin elevated and adiponectin low to low normal. Elevated resistin, high-normal to elevated TNFα, and elevated IL-6 levels. See Table 2 for figures of cholesterol, HDL, LDL.	

*NR* not reported

*No studies reported muscle mass outcomes

### Study quality

The assessment of study quality was brief (using a modified Newcastle–Ottawa scale with 8 very simple criteria). As shown in Table 4, apart from a lack of blinding of outcome assessors (not present in any study), most studies fulfilled most criteria.

**Table 4:** Quality of included studies (excluding case reports/series)

Reference	[26]	[25]	[18]	[19]	[23]	[24]	[27]	[1]	[20]	[21]	[10]	[22]
Study design*	1	1	2	2	3	3	4	2	4	4	4	4
1. Study groups	*N/A*	*N/A*	**Y**	**Y**	*N/A*	*N/A*	*N/A*	*N*	**Y**	**Y**	**Y**	**Y**
2. Attrition	*N/A*	*N/A*	*N*	**Y**	*N/A*	*N/A*	*N/A*	*NR*	**Y**	**Y**	*NR*	**Y**
3. Exposure measure	**Y**	**Y**	**Y**	**Y**	**Y**	**Y**	**Y**	**Y**	**Y**	**Y**	**Y**	**Y**
4. Outcome measure	**Y**	**Y**	**Y**	**Y**	**Y**	**Y**	**Y**	**Y**	**Y**	**Y**	**Y**	**Y**
5. Investigators blinded	*N*	*N*	*N*	*N*	*N*	*N*	*N*	*N*	*N*	*NR*	*N*	*N*
6. Confounders identified	**Y**	**Y**	**Y**	**Y**	**Y**	**Y**	**Y**	**Y**	**Y**	*N*	**Y**	**Y**
7. Statistical adjustment	**Y**	*N*	**Y**	**Y**	**Y**	**Y**	**Y**	**Y**	**Y**	*N*	*N*	**Y**
8. Funding source	*N*	**Y**	**Y**	**Y**	**Y**	*N*	**Y**	**Y**	**Y**	**Y**	**Y**	**Y**

Criteria based on the Newcastle–Ottawa scale [17]

*NR* not reported

*(1) Single group, (2) cross-sectional (controlled), (3) before and after (single group), (4) retrospective cohort

### Outcome data

Table 2 provides the outcome data for the controlled and uncontrolled studies. Outcomes for the case reports are included in Table 3. Due to heterogeneity within included studies (especially in terms of outcomes), we did not synthesize the data or perform any meta-analysis.

### Changes in body composition

The body composition results of the studies are reported in Table 2 and briefly summarized below.

#### Total body fat

There was little evidence of differences in total fat/weight between HSCT + TBI groups and healthy controls, population norms or short stature controls. Nysom et al. found significantly lower BMI compared to healthy controls [20]. Wei et al. also found significantly lower BMI and fat mass index, but this was compared to obese controls [10, 22]. Three studies found significantly higher body fat: body fat % compared to short stature controls [19] and healthy controls [20] and whole body fat mass *z* score compared to reference controls [1]. Data from Adachi et al. [26] suggests BMI may be lower than leukaemic controls with no TBI, and, although significance could not be tested, within the normal range for age.

#### Central adiposity

Most of the studies which measured central adiposity found significantly higher central adiposity for HSCT + TBI groups compared to leukaemic controls and non-leukaemic (obese/short stature/healthy) controls. Evidence from four studies found significant differences for lower waist-to-hip ratios and higher android-gynoid fat ratios compared to leukaemic controls and for higher waist circumference/waist–height ratio, greater trunk fat % and visceral fat %, compared to non-obese non-leukaemic controls [1, 19, 22, 118]. One study found evidence of significant differences for lower waist circumference/waist–height ratio, higher visceral fat % and higher visceral fat to total/subcutaneous fat ratios compared to obese non-leukaemic controls [10].

High waist-to-hip ratio was associated with features of metabolic syndrome in one study [22], and visceral fat % was associated with insulin resistance in another [1].

#### Adipose tissue function

All three studies which measured adipose tissue function found significant differences for HSCT + TBI groups compared to leukaemic controls and some to non-leukaemic controls. Compared to leukaemic controls, adiponectin was lower, leptin higher, triglycerides higher and high-density lipoprotein (HDL) lower [10, 18, 22]. The only difference compared to non-leukaemic controls (obese) was for raised triglycerides [22]. Lower adiponectin and HDL levels were more common in those with insulin resistance [1, 18].

#### Muscle mass

Four studies measured muscle mass (fat free/lean mass, muscle density), and all found significantly lower muscle mass for HSCT + TBI groups compared to healthy/obese controls and in HSCT + TBI patients compared with findings before HSCT + TBI [1, 10, 19, 27]. Wei et al. [10] found limited evidence for lower fat-free mass index compared to leukaemic controls. Lean mass/height^2^ was lower in females [27].

#### Muscle function

For HSCT + TBI groups compared to leukaemic controls, Taskinen et al. [21] found significant differences in some physical performance tests but not others, and Chow et al. found lower physical activity scores [18]. Davis et al. found some increase in strength following an exercise intervention [23].

### Association of body composition changes with metabolic status

Some studies commented on associations of body composition outcomes with the presence of features of metabolic syndrome. Associations with metabolic syndrome/insulin resistance were found with:

- Whole body fat mass [1]
- Waist-to-hip ratio and waist-to-height ratio [22]
- Subcutaneous adipose tissue, visceral adipose tissue [1]
- Lower adiponectin levels [25]
- Lower HDL [25]

### Potential factors modifying impact of HSCT on body composition

Although not an aim of this review, most studies reported on certain factors which may impact the relationship between HSCT and body composition, in particular graft versus host disease (GVHD), growth hormone deficiency and cranial radiation. This section briefly reports these results.

#### GVHD and treatment

Most studies reported the number of participants with GVHD, which varied from 0 to 61.5%. However, there was wide variability in reporting this and the details, i.e. whether acute or chronic. This is not a primary focus for this review. One study found that GVHD was predictive of underweight post-HSCT, and extensive chronic GVHD was predictive of lower BMI, but this was an uncontrolled study [27]. Three studies reported that GVHD or glucocorticoid treatment was not associated with body composition (cytokine levels [18]; marrow adipose tissue, any measures of adiposity or lean mass [1]; or whole-body % fat *z* score [20]).

#### GH

Two studies found an association of GH status with fat mass index (FMI) [10, 19] and gynoid fat% [10], but not with fat-free mass index (FFMI) [19], and other studies found no associations with body composition (cytokine levels [18], adiponectin [10], central fat [10] or different fat deposits from magnetic resonance imaging [10]).

#### Cranial radiation

Some studies explored the association of cranial radiation with body composition and found differences in BMI and whole body fat [20] but not in cytokine levels [18] or cardiometabolic traits [18].

#### Age at/time since HSCT

The studies showed mixed results regarding the relationship between time since HSCT and body composition. Age at HSCT was not associated with body composition in two studies (adiposity or lean mass [1] or whole-body % fat *z* score [20]).Two studies found no association (components of the metabolic syndrome [22], whole-body % fat *z* score [20] or measures of adiposity [1]) but did find a negative association with HDL [22] and adiponectin levels [10].

### Interventions to ameliorate changes in body composition

Only one study included an intervention [23]. The intervention (a 6-month programme of supervised, combined resistance and aerobic exercise) significantly improved aerobic fitness, insulin resistance and quality of life, although there were no changes in body composition. The authors concluded that the intervention had a metabolic training effect on muscle.

### Case reports

Table 3 presents characteristics and body composition data from the eleven cases reported in the eight case reports/series [28–35]. Seven had ALL and four AML; ten were female and one male. The cases were followed up an average of 11 years after HSCT. Nine of the eleven cases had GVHD and most had multiple complications/other diagnoses.

The data reported in the case reports/series presents a phenotype of lipodystrophy in leukaemic HSCT TBI patients which appears well described. All the cases were under- or normal weight based on their BMI (range 12.2 to 23.1) but showed clinical evidence of lipodystrophy with reduced fat in the limbs and gluteal region and increased fat centrally and in the face, with abdominal distension. Dyslipidaemia was noted in many cases, with elevated fasting triglycerides of between 332 and 927 mg/dL (3.75–10.5 mmol/L) (normal range < 150 mg/dL or < 1.7 mmol/L) but seemingly normal leptin levels of 3.5–7.4 ng/mL (normal range for females 8.8 + SEM 2.10 ng/mL [36]). Only one case report mentioned muscle function (limited range of motion and poor muscle tone); none of the reports mentioned muscle mass changes.

## Discussion

This review has found evidence that following HSCT with TBI as treatment for leukaemia in CTYA before the age of 25 years, there is remodelling of adipose tissue earlier than would be expected for age and an extreme phenotype of overt lipodystrophy. There is also some evidence for frailty and a reduction in muscle effectiveness/bulk/strength. These changes are associated with evidence for metabolic disadvantage which contributes to the risk of cardiovascular disease, particularly as abdominal obesity has been shown to be a risk factor for insulin resistance and impaired glucose tolerance following HSCT [37]. Although the literature is heterogeneous, limiting the conclusions we can draw, other studies of wider populations (not just leukaemia or not all TBI; excluded from our review) confirm this phenotype—for example reduced lean mass/increased fat mass for height in HSCT patients [5], increased abdominal adiposity and hypertriglyceridemia [38] and increased sarcopenia [39].

Although the mechanisms for how HSCT with TBI affects body composition was a not a focus for this review, some studies mentioned factors which may additionally impact on body composition, including GVHD, growth hormone deficiency and cranial radiation. There is a need to understand why the changes in muscle and fat occur following HSCT.

### Clinical implications

The 2012 guidelines on screening and preventive practices for long-term survivors of HSCT [40] include recommendations for early treatment of cardiovascular risk factors such as diabetes, hypertension and dyslipidaemia and education and counselling on healthy lifestyle (regular exercise, maintaining healthy weight, no smoking, dietary counselling). Griffith et al. [41] also provide detailed recommendations on the evaluation and management of dyslipidaemia in HSCT patients. Nevertheless, the key issue is whether any interventions can be shown to help mitigate or even reverse the adverse changes to body composition and the apparent link to the cardiometabolic risk.

We only identified one study which tested an intervention [23]; whilst this showed effects on fitness, insulin resistance and quality of life, it did not demonstrate any effects on body composition. Studies on wider populations have found some positive effects for exercise and nutritional supplementation during or after TBI: increased body mass and BMI, partly mediated by an increase in fat-free mass [42]; improved muscular strength and endurance performance [43]; increased fat free mass and decreased body fat [44]; and improved muscle mass [45].

Many conventional weight loss techniques would not be appropriate in this population as patients after HSCT with TBI are not overweight and nonspecific weight loss could exacerbate their situation due to further loss of muscle mass. Although one study demonstrated the feasibility and acceptability of a strength-training intervention for patients undergoing HSCT [46], it is possible that exercise benefits may be limited, due to reduced muscle mass. There is therefore a need to develop innovative interventions to improve the muscle function and metabolic effectiveness of long-term survivors of HSCT with TBI in the CTYA years, perhaps utilizing dietary supplements and targeted forms of physical activity.

## Limitations

There are limitations to this review. As a restricted systematic review, the screening of articles was less comprehensive than for a systematic review and there is a chance that eligible papers were excluded. We have included in Online Resource 2 lists of excluded papers. Responses from key authors in the field confirmed that we had identified most relevant studies. Searching only one database may have meant we missed relevant papers. However, this methodology is acceptable for a restricted systematic review, and we also attempted to identify grey literature and did not limit by date or language [15].

This review did not aim to identify potential mechanisms leading to body composition changes, so did not systematically collect data on associations with factors such as GVHD, additional/prior radiotherapy, e.g. to the central nervous system or abdomen, or endocrine status.

Most of the included studies were not designed with body composition as their primary outcome, meaning our final sample covered a very diverse range of study designs and outcomes, making data synthesis difficult. The variation in demographics of the study populations makes it difficult to compare outcome data to population norms.

The studies included also have their own limitations. Studies all used convenience samples, with very little information reported on those who did not volunteer to participate. We are therefore unable to comment on how representative our results are to the general leukaemia HSCT with TBI population. Few studies reported participants’ ethnicity or were mostly composed of those with white ethnicities, which is a potential deficiency given that ethnicity can affect body composition and the risk of metabolic disruption when abnormal [47].

## Conclusion

This review has found evidence that allogeneic HSCT with TBI for CTYA leukaemia results in remodelling of adipose tissue earlier than is expected for age, with the extreme phenotype of overt lipodystrophy. There is also some evidence for a reduction in muscle effectiveness/bulk/strength. These changes mirror those seen with normal ageing and appear to associate with measures of early cardiovascular morbidity. Innovative interventions are needed to determine if changes in muscle and adipose function and metabolic effectiveness can be reversed/mitigated at any age after HSCT, perhaps utilizing dietary manipulation and/or targeted exercise and activity interventions.

## Electronic supplementary material

ESM 1 (PDF 135 kb)

ESM 2 (PDF 197 kb)
