# Supplementary material for: Body composition after allogeneic haematopoietic cell transplantation/total body irradiation in children and young people: a restricted systematic review
Source: J Cancer Surviv. 2020 Jul 18;14(5):624–42. doi: 10.1007/s11764-020-00871-1 (PMC7473918; doi:10.1007/s11764-020-00871-1)
Supplement: Supplementary file 1 — (PDF 135 kb) [file 11764_2020_871_MOESM1_ESM.pdf]

Supplementary file 1: Search terms

Changes in body composition after allogeneic haematopoietic stem cell transplantation (HSCT) with total body irradiation (TBI) for treatment of leukaemia in children, teenagers and young adults (CTYA): a restricted systematic review

Submitted to: Journal of Cancer Surviorship

Dr Ava Lorenc, Prof Julian Hamilton-Shield, Dr Rachel Perry, Prof Michael Stevens on behalf of the CTYA HSCT Adipose and Muscle late effects working group*

*Corresponding author

Tel: 07796 146440

m.stevens@bristol.ac.uk

Medline:

1. Lipodystro*.ti,ab.
2. Exp lipodystrophy/
3. (visceral adj1 fat).ti,ab.
4. (central adj1 fat).ti,ab.
5. Exp body fat distribution/
6. (fat adj1 distribut*).ti,ab.
7. (fat adj1 compart*).ti,ab.
8. Exp Abdominal Fat/
9. (abdominal adj1 fat).ti,ab.
10. (abdominal adj1 obes*).ti,ab.
11. (Body adj1 composition).ti,ab.
12. exp body composition/
13. exp Waist-Hip Ratio/
14. (waist adj1 hip adj1 ratio).ti,ab.
15. exp body mass index/
16. (body adj1 mass adj1 index).ti,ab.
17. BMI.ti,ab.
18. Frailty.ti,ab.
19. Exp Frailty/
20. Sarcopen*.ti,ab.
21. Exp Muscular atrophy/
22. (Muscle adj1 mass).ti,ab.
23. Body Height/
24. Height.ti,ab.

25. 1 or 2 or 3 or 4 or 5 or 6 or 7 or 8 or 9 or 10 or 11 or 12 or 13 or 14 or 15 or 16 or 17 or 18 or 19 or 20 or 21 or 22 or 23 or 24

26. HSCT.ti,ab.
27. HCT.ti,ab.
28. (h?ematopoietic adj1 stem adj1 cell adj1 transplant*).ti,ab.
29. (h?ematopoietic adj1 cell adj1 transplant*).ti,ab.
30. stem cell transplant.ti,ab.
31. (stem adj1 cell adj1 transplant*).ti,ab.
32. Exp Stem Cell Transplantation/
33. Bone Marrow Transplantation/
34. BMT.ti,ab.
35. (bone adj1 marrow adj1 transplant*).ti,ab.
36. h?ematocrit.ti,ab.

37. 26 or 27 or 28 or 29 or 30 or 31 or 32 or 33 or 34 or 35
38. 37 NOT 36

39. 25 AND 38

40. exp Animals, Laboratory/
41. exp Animal Experimentation/
42. exp models, Animal/
43. Rodentia/
44. (rat* or mouse or mice).ti.
45. exp in vitro techniques/
46. in vitro.ti
47. pre-clinical.ti,ab.
48. 38 or 39 or 40 or 41 or 42 or 43 or 44 or 45

49. 39 NOT 48

Google scholar:

(lipodystrophy OR fat OR waist-hip OR BMI OR body mass index OR frailty OR sarcopenia OR muscle OR height) AND (HSCT or stem cell transplant OR bone marrow transplant OR BMT)
