# Supplementary material for: Body composition after allogeneic haematopoietic cell transplantation/total body irradiation in children and young people: a restricted systematic review
Source: J Cancer Surviv. 2020 Jul 18;14(5):624–42. doi: 10.1007/s11764-020-00871-1 (PMC7473918; doi:10.1007/s11764-020-00871-1)
Supplement: Supplementary file 2 — (PDF 197 kb) [file 11764_2020_871_MOESM2_ESM.pdf]

Supplementary file 2: Selected excluded studies

Changes in body composition after allogeneic haematopoietic stem cell transplantation (HSCT) with total body irradiation (TBI) for treatment of leukaemia in children, teenagers and young adults (CTYA): a restricted systematic review

Submitted to: Journal of Cancer Survivorship

Dr Ava Lorenc, Prof Julian Hamilton-Shield, Dr Rachel Perry, Prof Michael Stevens on behalf of the CTYA HSCT Adipose and Muscle late effects working group*

*Corresponding author

Tel: 07796 146440

m.stevens@bristol.ac.uk

The following papers did not meet the inclusion criteria for this review (they were excluded at the full text screening stage) but may nevertheless include relevant information on the topic. We provide them here for reference.

Body composition outcomes were measured but are not the main focus of the study (n=27).

Afify, Z., P. J. Shaw, A. Clavano-Harding and C. T. Cowell (2000). "Growth and endocrine function in children with acute myeloid leukaemia after bone marrow transplantation using busulfan/cyclophosphamide." Bone Marrow Transplantation **25**(10): 1087-1092.

Alter, C. A., P. S. Thornton, S. M. Willi, N. Bunin and T. Moshang, Jr. (1996). "Growth in children after bone marrow transplantation for acute myelogenous leukemia as compared to acute lymphocytic leukemia." Journal of Pediatric Endocrinology & Metabolism **9**(1): 51-57.

Aplenc, R., M. J. Zhang, L. Sung, X. Zhu, V. T. Ho, K. Cooke, C. Dvorak, G. Hale, L. M. Isola, H. M. Lazarus, P. L. McCarthy, R. Olsson, M. Pulsipher, M. C. Pasquini, N. Bunin, C. f. I. B. Regimen-Related Toxicity Working Committee and R. Marrow Transplant (2014). "Effect of body mass in children with hematologic malignancies undergoing allogeneic bone marrow transplantation." Blood **123**(22): 3504-3511.

Bakker, B., G. G. Massa, W. Oostdijk, M. H. Van Weel-Sipman, J. M. Vossen and J. M. Wit (2000). "Pubertal development and growth after total-body irradiation and bone marrow transplantation for haematological malignancies." European Journal of Pediatrics **159**(1-2): 31-37.

Bakker, B., W. Oostdijk, R. B. Geskus, W. H. Stokvis-Brantsma, J. M. Vossen and J. M. Wit (2006). "Patterns of growth and body proportions after total-body irradiation and hematopoietic stem cell transplantation during childhood." Pediatric Research **59**(2): 259-264.

Bakker, B., W. Oostdijk, R. B. Geskus, W. H. Stokvis-Brantsma, J. M. Vossen and J. M. Wit (2007). "Growth hormone (GH) secretion and response to GH therapy after total body irradiation and haematopoietic stem cell transplantation during childhood." Clinical Endocrinology **67**(4): 589-597.

Bernard, F., P. Bordigoni, M. C. Simeoni, V. Barlogis, A. Contet, A. Loundou, I. Thuret, B. Leheup, H. Chambost, B. Play, P. Auquier and G. Michel (2009). "Height growth during adolescence and final height after haematopoietic SCT for childhood acute leukaemia: the impact of a conditioning regimen with BU or TBI." Bone Marrow Transplantation **43**(8): 637-642.

Bushhouse, S., N. K. Ramsay, O. H. Pescovitz, T. Kim and L. L. Robison (1989). "Growth in children following irradiation for bone marrow transplantation." American Journal of Pediatric Hematology/Oncology **11**(2): 134-140.

Choi, Y. J., S. Y. Park, W. K. Cho, J. W. Lee, K. S. Cho, S. H. Park, S. H. Hahn, M. H. Jung, N. G. Chung, B. Cho, B. K. Suh and H. K. Kim (2013). "Factors related to decreased bone mineral density in childhood cancer survivors." Journal of Korean Medical Science **28**(11): 1632-1638.

Cohen, A., A. Rovelli, M. T. Van-Lint, C. Uderzo, A. Morchio, C. Pezzini, G. Masera, A. Bacigalupo and C. Romano (1996). "Final height of patients who underwent bone marrow transplantation during childhood." Archives of Disease in Childhood **74**(5): 437-440.

Couto-Silva, A. C., C. Trivin, H. Esperou, J. Michon, A. Baruchel, J. C. Souberbielle and R. Brauner (2010). "Bone markers after total body irradiation in childhood." Bone Marrow Transplantation **45**(3): 437-441.

Eissa, H. M., L. Lu, M. Baassiri, N. Bhakta, M. J. Ehrhardt, B. M. Triplett, D. M. Green, D. A. Mulrooney, L. L. Robison, M. M. Hudson and K. K. Ness (2017). "Chronic disease burden and frailty in survivors of childhood HSCT: a report from the St. Jude Lifetime Cohort Study." Blood Advances **1**(24): 2243-2246.

Friedman, D. N., P. Hilden, C. S. Moskowitz, M. Suzuki, F. Boulad, N. A. Kernan, S. L. Wolden, K. C. Oeffinger and C. A. Sklar (2017). "Cardiovascular Risk Factors in Survivors of Childhood Hematopoietic Cell Transplantation Treated with Total Body Irradiation: A Longitudinal Analysis." Biology of Blood & Marrow Transplantation **23**(3): 475-482.

Galletto, C., A. Gliozzi, D. Nucera, N. Bertorello, E. Biasin, A. Corrias, P. Chiabotto, F. Fagioli and C. Guiot (2014). "Growth impairment after TBI of leukemia survivors children: a model- based investigation." Theoretical Biology & Medical Modelling **11**: 44.

Holm, K., K. Nysom, M. H. Rasmussen, H. Hertz, N. Jacobsen, N. E. Skakkebaek, S. Krabbe and J. Muller (1996). "Growth, growth hormone and final height after BMT. Possible recovery of irradiation-induced growth hormone insufficiency." Bone Marrow Transplantation **18**(1): 163-170.

Hovi, L., J. Rajantie, M. Perkkio, K. Sainio, I. Sipila and M. A. Siimes (1990). "Growth failure and growth hormone deficiency in children after bone marrow transplantation for leukemia." Bone Marrow Transplantation **5**(3): 183-186.

Huma, Z., F. Boulad, P. Black, G. Heller and C. Sklar (1995). "Growth in children after bone marrow transplantation for acute leukemia." Blood **86**(2): 819-824.

Iestra, J. A., W. E. Fibbe, A. H. Zwinderman, J. A. Romijn and D. Kromhout (1999). "Parenteral nutrition following intensive cytotoxic therapy: an exploratory study on the need for parenteral nutrition after various treatment approaches for haematological malignancies." Bone Marrow Transplantation **23**(9): 933-939.

Leiper, A. D., R. Stanhope, T. Lau, D. B. Grant, H. Blacklock, J. M. Chessells and P. N. Plowman (1987). "The effect of total body irradiation and bone marrow transplantation during childhood and adolescence on growth and endocrine function." British Journal of Haematology **67**(4): 419-426.

Leung, W., M. Hudson, Y. Zhu, G. K. Rivera, R. C. Ribeiro, J. T. Sandlund, L. C. Bowman, W. E. Evans, L. Kun and C. H. Pui (2000). "Late effects in survivors of infant leukemia." Leukemia **14**(7): 1185-1190.

Leung, W., M. M. Hudson, D. K. Strickland, S. Phipps, D. K. Srivastava, R. C. Ribeiro, J. E. Rubnitz, J. T. Sandlund, L. E. Kun, L. C. Bowman, B. I. Razzouk, P. Mathew, P. Shearer, W. E. Evans and C. H. Pui (2000). "Late effects of treatment in survivors of childhood acute myeloid leukemia." Journal of Clinical Oncology **18**(18): 3273-3279.

Mostoufi-Moab, S., J. P. Ginsberg, N. Bunin, B. Zemel, J. Shults and M. B. Leonard (2012). "Bone density and structure in long-term survivors of pediatric allogeneic hematopoietic stem cell transplantation." Journal of Bone & Mineral Research **27**(4): 760-769.

Nysom, K., K. Holm, K. F. Michaelsen, H. Hertz, N. Jacobsen, J. Muller and C. Molgaard (2000). "Bone mass after allogeneic BMT for childhood leukaemia or lymphoma." Bone Marrow Transplantation **25**(2): 191-196.

Papadimitriou, A., M. Urena, G. Hamill, R. Stanhope and A. D. Leiper (1991). "Growth hormone treatment of growth failure secondary to total body irradiation and bone marrow transplantation." Archives of Disease in Childhood **66**(6): 689-692.

Quek, S. C., K. Y. Loke, T. C. Quah, S. K. Lam and B. W. Lee (1992). "Long term endocrine complications of acute leukemia therapy--a case report." Journal of the Singapore Paediatric Society **34**(3-4): 214-216.

Thuret, I., G. Michel, H. Carla, H. Chambost, D. Blaise, F. Demeocq and D. Maraninchi (1995). "Long-term side-effects in children receiving allogeneic bone marrow transplantation in first complete remission of acute leukaemia." Bone Marrow Transplantation **15**(3): 337-341.

Uderzo, C., A. Locasciulli, A. Rovelli, M. R. Rossi, M. Jankovic, L. Adamoli, M. Bonomi, A. Balduzzi, A. Biondi, R. Schiro and et al. (1992). "Bone marrow transplantation for childhood leukemia: five years' experience in a pediatric hematology center." Haematologica **77**(3): 257-264.

Included leukaemia but also other health conditions (n=37)

Anderson, L., D. Schmidt, K. Bingen, M. J. Kupst and A. Warwick (2009). "Growth velocity in pediatric bone marrow transplantation: significance of donor type and treatment factors." Journal of Pediatric Oncology Nursing **26**(6): 377-382.

Azarnoush, S., B. Bruno, L. Beghin, D. Guimber, B. Nelken, I. Yakoub-Agha and D. Seguy (2012). "Enteral nutrition: a first option for nutritional support of children following allo-SCT?" Bone Marrow Transplantation **47**(9): 1191-1195.

Baena-Gomez, M. A., M. J. de la Torre Aguilar, M. D. Mesa, F. J. Llorente-Cantarero, J. L. Perez Navero and M. Gil-Campos (2013). "Effects of parenteral nutrition formulas on plasma lipid profile in children with bone marrow transplantation." Annals of Nutrition & Metabolism **63**(1-2): 103-110.

Barnum, J. L., A. Petryk, L. Zhang, T. E. DeFor, K. S. Baker, J. Steinberger, B. Nathan, J. E. Wagner and M. L. MacMillan (2016). "Endocrinopathies, Bone Health, and Insulin Resistance in Patients with Fanconi Anemia after Hematopoietic Cell Transplantation." Biology of Blood & Marrow Transplantation **22**(8): 1487-1492.

Bizzarri, C., R. M. Pinto, S. Ciccone, L. P. Brescia, F. Locatelli and M. Cappa (2015). "Early and progressive insulin resistance in young, non-obese cancer survivors treated with hematopoietic stem cell transplantation." Pediatric Blood & Cancer **62**(9): 1650-1655.

Bouma, S., M. Peterson, E. Gatza and S. W. Choi (2016). "Nutritional status and weakness following pediatric hematopoietic cell transplantation." Pediatric Transplantation **20**(8): 1125-1131.

Bresters, D., I. C. van Gils, W. J. Kollen, L. M. Ball, W. Oostdijk, J. G. van der Bom and R. M. Egeler (2010). "High burden of late effects after haematopoietic stem cell transplantation in childhood: a single-centre study." Bone Marrow Transplant **45**(1): 79-85.

Bulley, S., A. Gassas, L. L. Dupuis, R. Aplenc, J. Beyene, M. L. Greenberg, J. J. Doyle and L. Sung (2008). "Inferior outcomes for overweight children undergoing allogeneic stem cell transplantation." British Journal of Haematology **140**(2): 214-217.

Cicognani, A., E. Cacciari, A. Pession, A. Pasini, R. De Iasio, M. Gennari, P. Alvisi and P. Pirazzoli (1999). "Insulin-like growth factor-I (IGF-I) and IGF-binding protein-3 (IGFBP-3) concentrations compared to stimulated growth hormone (GH) in the evaluation of children treated for malignancy." Journal of Pediatric Endocrinology & Metabolism **12**(5): 629-638.

Cohen, A., T. Duell, G. Socie, M. T. van Lint, M. Weiss, A. Tichelli, A. Rovelli, J. F. Apperley, P. Ljungman and H. J. Kolb (1999). "Nutritional status and growth after bone marrow transplantation (BMT) during childhood: EBMT Late-Effects Working Party retrospective data. European Group for Blood and Marrow Transplantation." Bone Marrow Transplantation **23**(10): 1043-1047.

Couto-Silva, A. C., C. Trivin, H. Esperou, J. Michon, A. Baruchel, P. Lemaire and R. Brauner (2006). "Final height and gonad function after total body irradiation during childhood." Bone Marrow Transplantation **38**(6): 427-432.

Feng, Y., L. Y. Pan, L. Y. Shen, P. P. Chang, B. H. Zhang and L. Hong (2018). "Changes in body composition in children with acute graft-versus-host disease within the first 100 days after hematopoietic stem cell transplantation." European Journal of Clinical Nutrition **72**(8): 1167-1175.

Frisk, P., J. Arvidson, M. Larsson and T. Naessen (2012). "Risk factors for cardiovascular disease are increased in young adults treated with stem cell transplantation during childhood." Pediatric Transplantation **16**(4): 385-391.

Giorgiani, G., M. Bozzola, F. Locatelli, P. Picco, M. Zecca, M. Cisternino, S. Dallorso, F. Bonetti, G. Dini, C. Borrone and et al. (1995). "Role of busulfan and total body irradiation on growth of prepubertal children receiving bone marrow transplantation and results of treatment with recombinant human growth hormone." Blood **86**(2): 825-831.

Guieze, R., R. Lemal, A. Cabrespine, E. Hermet, O. Tournilhac, C. Combal, J. O. Bay and C. Bouteloup (2014). "Enteral versus parenteral nutritional support in allogeneic haematopoietic stem-cell transplantation." Clinical Nutrition **33**(3): 533-538.

Ishida, Y., M. Honda, S. Ozono, J. Okamura, K. Asami, N. Maeda, N. Sakamoto, H. Inada, T. Iwai, K. Kamibeppu, N. Kakee and K. Horibe (2010). "Late effects and quality of life of childhood cancer survivors: part 1. Impact of stem cell transplantation." International Journal of Hematology **91**(5): 865-876.

Jung, M. H., K. S. Cho, J. W. Lee, N. G. Chung, B. Cho, B. K. Suh, H. K. Kim and B. C. Lee (2009). "Endocrine complications after hematopoietic stem cell transplantation during childhood and adolescence." Journal of Korean Medical Science **24**(6): 1071-1077.

Jung, M. H., K. S. Cho, J. W. Lee, N. G. Chung, B. Cho, B. K. Suh, H. K. Kim and B. C. Lee (2009). "Endocrine Complications after Hematopoietic Stem Cell Transplantation during Childhood and Adolescence." J Korean Med Sci **24**(6): 1071-1077.

Legault, L. and Y. Bonny (1999). "Endocrine complications of bone marrow transplantation in children." Pediatric Transplantation **3**(1): 60-66.

Majhail, N. S., T. R. Challa, D. A. Mulrooney, K. S. Baker and L. J. Burns (2009). "Hypertension and Diabetes Mellitus in Adult and Pediatric Survivors of Allogeneic Hematopoietic Cell Transplantation." Biology of Blood and Marrow Transplantation **15**(9): 1100-1107.

Neville, K. A., R. J. Cohn, K. S. Steinbeck, K. Johnston and J. L. Walker (2006). "Hyperinsulinemia, impaired glucose tolerance, and diabetes mellitus in survivors of childhood cancer: prevalence and risk factors." Journal of Clinical Endocrinology & Metabolism **91**(11): 4401-4407.

Oberg, A., M. Genberg, A. Malinovschi, H. Hedenstrom and P. Frisk (2018). "Exercise capacity in young adults after hematopoietic cell transplantation in childhood." American Journal of Transplantation **18**(2): 417-423.

Papadopoulou, A., M. D. Williams, P. J. Darbyshire and I. W. Booth (1998). "Nutritional support in children undergoing bone marrow transplantation." Clinical Nutrition **17**(2): 57-63.

Paris, C., L. Yates, P. Lama, A. J. Zepeda, D. Gutierrez and J. Palma (2012). "Evaluation of metabolic syndrome after hematopoietic stem cell transplantation in children and adolescents." Pediatric Blood & Cancer **59**(2): 306-310.

Pine, M., L. Wang, F. E. Harrell, Jr., C. Calder, B. Manes, M. Evans, J. Domm and H. Frangoul (2011). "The effect of obesity on outcome of unrelated cord blood transplant in children with malignant diseases." Bone Marrow Transplantation **46**(10): 1309-1313.

Rodgers, C., M. A. Gregurich and M. Hockenberry (2012). "Lipid profiles of pediatric hematopoietic stem cell transplant survivors." Journal of Pediatric Oncology Nursing **29**(2): 63-69.

Ruble, K., M. Hayat, K. J. Stewart and A. Chen (2012). "Body composition after bone marrow transplantation in childhood." Oncology Nursing Forum **39**(2): 186-192.

Shalitin, S., L. Pertman, M. Yackobovitch-Gavan, I. Yaniv, Y. Lebenthal, M. Phillip and J. Stein (2018). "Endocrine and Metabolic Disturbances in Survivors of Hematopoietic Stem Cell Transplantation in Childhood and Adolescence." Hormone research in paediatrics **89**(2): 108-121.

Slater, M. E., J. Steinberger, J. A. Ross, A. S. Kelly, E. J. Chow, I. H. Koves, P. Hoffmeister, A. R. Sinaiko, A. Petryk, A. Moran, J. Lee, L. S. Chow and K. S. Baker (2015). "Physical Activity, Fitness, and Cardiometabolic Risk Factors in Adult Survivors of Childhood Cancer with a History of Hematopoietic Cell Transplantation." Biology of Blood & Marrow Transplantation **21**(7): 1278-1283.

Taskinen, M., M. Lipsanen-Nyman, A. Tiitinen, L. Hovi and U. M. Saarinen-Pihkala (2007). "Insufficient growth hormone secretion is associated with metabolic syndrome after allogeneic stem cell transplantation in childhood." Journal of Pediatric Hematology/Oncology **29**(8): 529-534.

Taskinen, M., U. M. Saarinen-Pihkala, L. Hovi and M. Lipsanen-Nyman (2000). "Impaired glucose tolerance and dyslipidaemia as late effects after bone-marrow transplantation in childhood." Lancet **356**(9234): 993-997.

Uderzo, C., E. Biagi, A. Rovelli, A. Balduzzi, R. Schiro, D. Longoni, C. Arrigo, B. Nicolini, L. Placa, A. Da Prada, L. Mascaretti, G. Giltri, S. Galimberti, M. G. Valsecchi, A. Locasciulli and G. Masera (2000). "Bone marrow transplantation for childhood hematological disorders: a global pediatric approach in a twelve year single center experience." Pediatria Medica e Chirurgica **21**(4): 157-163.

Wegener, D., P. Lang, F. Paulsen, N. Weidner, D. Zips, M. Ebinger, U. Holzer, M. Doring, O. Basu, B. Gruhn, A. Wittig, H. M. Teltschik, R. Handgretinger and F. Heinzelmann (2019). "Immunosuppressive Total Nodal Irradiation-Based Reconditioning Regimens After Graft Rejection or Graft Failure in Pediatric Patients Treated With Myeloablative Allogeneic Hematopoietic Cell Transplantation." International Journal of Radiation Oncology, Biology, Physics **104**(1): 137-143.

Wingard, J. R., L. P. Plotnick, C. S. Freemer, M. Zahurak, S. Piantadosi, D. F. Miller, H. M. Vriesendorp, A. M. Yeager and G. W. Santos (1992). "Growth in children after bone marrow transplantation: busulfan plus cyclophosphamide versus cyclophosphamide plus total body irradiation." Blood **79**(4): 1068-1073.

Wojcik, D., E. Barg, E. Niedzielska, A. Doroszko, K. Kalwak and W. Pietras (2006). "[Analysis of some risk factors for abnormal growth velocity in children treated with haematopoetic stem cell transplantation]." Medycyna Wieku Rozwojowego **10**(3 Pt 1): 841-848.

Wood, R. M. (1990). "Growth patterns in pediatric bone marrow transplant patients." Journal of Pediatric Nursing **5**(4): 252-258.

Yokoyama, S., T. Fujimoto, T. Mitomi, M. Yabe, H. Yabe and S. Kato (1989). "Use of total parenteral nutrition in pediatric bone marrow transplantation." Nutrition **5**(1): 27-30.

Included both autologous and allogeneic HSCT

Arvidson, J., G. Lonnerholm, T. Tuvemo, K. Carlson, B. Lannering and T. Lonnerholm (2000). "Prepubertal growth and growth hormone secretion in children after treatment for hematological malignancies, including autologous bone marrow transplantation." Pediatric Hematology & Oncology **17**(4): 285-297.

Beauloye, V., M. Steffens, F. Zech, C. Vermylen and D. Maiter (2013). "Characterization of insulin resistance in young adult survivors of childhood acute lymphoblastic leukaemia and non-Hodgkin lymphoma." Clinical Endocrinology **78**(5): 790-798.

Frisk, P., S. M. Rossner, S. Norgren, J. Arvidson and J. Gustafsson (2011). "Glucose metabolism and body composition in young adults treated with TBI during childhood." Bone Marrow Transplantation **46**(10): 1303-1308.

Oudin, C., P. Auquier, Y. Bertrand, A. Contet, J. Kanold, N. Sirvent, S. Thouvenin, M. D. Tabone, P. Lutz, S. Ducassou, D. Plantaz, J. H. Dalle, V. Gandemer, S. Beliard, J. Berbis, C. Vercasson, V. Barlogis, A. Baruchel, G. Leverger and G. Michel

(2015). "Metabolic syndrome in adults who received hematopoietic stem cell transplantation for acute childhood leukemia: an LEA study." Bone Marrow Transplantation **50**(11): 1438-1444.

Tahrani, A. A., C. Cramp and P. Moulik (2006). "The development of non-insulin-dependent diabetes after total body irradiation and bone marrow transplantation in adolescence: a case report and literature review." Pediatric Diabetes **7**(3): 173-175.

Visentin, S., G. Michel, C. Oudin, B. Cousin, B. Gaborit, I. Abdesselam, M. Maraninchi, M. Nowicki, R. Valero, M. Guye, M. Bernard, P. Auquier, H. Chambost, M.-C. Alessi and S. Beliard (2019). "Lipodystrophy-like features after total body irradiation among survivors of childhood acute leukemia." Endocrine connections **8**(4): 349-359.
